# *Ganoderma lucidum*: Unutilized natural medicine and promising future solution to emerging diseases in Africa

**DOI:** 10.3389/fphar.2022.952027

**Published:** 2022-08-22

**Authors:** M. A. Oke, F. J. Afolabi, O. O. Oyeleke, T. A. Kilani, A. R. Adeosun, A. A. Olanbiwoninu, E. A. Adebayo

**Affiliations:** ^1^ Department of Pure and Applied Biology, Ladoke Akintola University of Technology, Ogbomoso, Nigeria; ^2^ Microbiology and Nanobiotechnology Laboratory, LAUTECH, Ogbomoso, Nigeria; ^3^ Mushrooms Department, National Biotechnology Development Centre, Ogbomoso, Nigeria; ^4^ Department of Biological Sciences, Ajayi Crowther University, Oyo, Nigeria

**Keywords:** Ganoderma lucidum, infectious disease, triterpenoids, polysaccharides, medicinal mushroom

## Abstract

*Ganoderma lucidum* is a well-known medicinal mushroom that has been used for the prevention and treatment of different ailments to enhance longevity and health specifically in China, Japan, and Korea. It was known as “God’s herb” in ancient China as it was believed to prolong life, enhance the youthful spirit and sustain/preserve vitality. *G. lucidum* is seldom collected from nature and is substantially cultivated on wood logs and sawdust in plastic bags or bottles to meet the international market demand. Both *in vitro* and *in vivo* studies on the copious metabolic activities of *G. lucidum* have been carried out. Varied groups of chemical compounds including triterpenoids, polysaccharides, proteins, amino acids, nucleosides, alkaloids, steroids, lactones, lectins, fatty acids, and enzymes with potent pharmacological activities have been isolated from the mycelia and fruiting bodies of *G. lucidum.* Several researchers have reported the abundance and diversification of its biological actions triggered by these chemical compounds. Triterpenoids and polysaccharides of *G. lucidum* have been reported to possess cytotoxic, hepatoprotective, antihypertensive, hypocholesterolemic, antihistaminic effects, antioxidant, antimicrobial, anti-inflammatory, hypoglycemic antiallergic, neuroprotective, antitumor, immunomodulatory and antiangiogenic activities. Various formulations have been developed, patented, and utilized as nutraceuticals, cosmeceuticals, and pharmaceuticals from *G. lucidum* extracts and active compounds. Thus, this review presents current updates on emerging infectious diseases and highlights the scope, dynamics, and advances in infectious disease management with a particular focus on *Ganoderma lucidum,* an unutilized natural medicine as a promising future solution to emerging diseases in Africa. However, details such as the chemical compound and mode of action of each bioactive against different emerging diseases were not discussed in this study.

## Introduction

Globally, the main cause of mortality and morbidity remains emerging infectious diseases due to the frequency of occurrence of new infections, re-occurrence of old infections, and the endemic nature of intractable infections (Holmes et al., 2017). This is a great challenge to Africa, achieving the goal of 2063 of a healthy continent, and has been the key impediment to the rate of development of countries in the continent (Holmes et al., 2017; Nkengasong and Tessema, 2020). Africa has the peak occurrence of communicable diseases in the world, while the majority of the estimated (10 million per-annual deaths) from infectious diseases occur in the continent (Hill-Cawthorne and Sorrell, 2016; Nkengasong and Tessema, 2020). Infectious diseases are generally caused by micro-organisms which may be bacteria, viruses, parasites, fungi etc. Seventy-five percent of these diseases are zoonotic and have been sources of serious health threats to the world dating back to ancient Egypt. A pathogenic microbial agent including bacteria, viruses, parasites, or fungi of which approximately 75% are zoonotic diseases that have jumped taxonomic lines to infect humans, causing grievous health challenges to the world since the days of ancient Egypt (WHO, 2005; Weber et al., 2016).

Infectious diseases like malaria, diarrhea, and *tuberculosis* have been reported among the top ten causes of death in developing countries (WHO, 2008) while the Acute Respiratory Syndrome (SARS) coronavirus 2 (COVID-19) has been reported lately as one of the world’s deadliest pandemics (Nueangnong et al., 2020; Di et al., 2021). It was reported on 4 January 2022 that COVID-19 has infected up to two hundred and eighty-one million eight hundred and eight thousand two hundred and seventy (281, 808, 270) persons globally with five million, four hundred and eleven thousand seven hundred and fifty-nine (5,411,759) deaths recorded. In Africa, seven million, one hundred and sixty-four thousand four hundred and eighty-five (7,164,485) infections were recorded while one hundred and fifty-five thousand six hundred and seventy-five (155,675) deaths mortalities were recorded (Makinde et al., 2022). Despite the lowest number of COVID-19 confirmed cases in Africa, Africa has a long history of severe and re-emerging infectious disease upsurges such as malaria and ebola (Fenollar and Mediannikov, 2018).

It has been estimated that over 227 million lives have been lost to infectious diseases and it is also documented to be responsible for an annual productivity loss of over US$800 billion yearly (WHO, 2019a). Economic, political, climatic, and environmental changes coupled with many more challenges already facing the continent have been ascribed to the dreadful cases or impact of infectious disease outbreaks in Africa (WHO, 2014). The ability to transmit infectious diseases from one person or species to another makes them more contagious, which may arise *via* one or more different ways such as physical interaction with affected individuals, airborne inhalation, vector-borne spread, and contaminated objects (Dorland, 2004).

In combating these diseases, a multifaceted approach ranging from drug/vaccine discovery and development, improved sanitation, and improved healthcare delivery at primary, secondary, and tertiary levels have been employed to varying degrees of success. Despite these efforts some of the defeated or successfully suppressed ones are resurfacing or have resurfaced in the latter part of the 20th century (Gardner et al., 2002; Holt et al., 2002; Fauci, 2006; Goel et al., 2006). Since the inception of the 21st century till date, a global increase in outbreaks of EIDs has been experienced with significant public health alarms; these include but are not limited to outbreaks of H1N1 swine flu in 2009, Ebola virus in 2013–2016, Severe Acute Respiratory Syndrome-related coronavirus (SARS-CoV) outbreak between 2003 and 2004, Middle East respiratory syndrome coronavirus (MERS-CoV) in 2012, and Zika virus from 2015 to 2016 (Kaner and Schaack, 2016; Aleanizy et al., 2017; Pereira et al., 2018).

A lot of factors such as rapid population increase, intense international travel, poor sanitation in congested cities, a substantial increase in the international food trade, poor practices of food preparation, continuous exposure to disease vectors and reservoirs, and climate change affect the types and proliferation of insect vectors and animal reservoirs. Other factors include poor public health institutions and infrastructures, natural species diversity, recombination and adaptations, uncontrolled application of pesticides and antimicrobial drugs and the use of virulent pathogens such as *Bacillus anthracis* and smallpox virus as agents of bioterrorism have been reported as contributing factors to disease emergence and re-emergence (Woolhouse and Gowtage-Sequeria, 2005; Vignier and Bouchaud, 2018). Hence, infectious diseases are certainly an unending threat to the whole world irrespective of race, colour, lifestyle, socioeconomic status, and ethnic background. They pose misery, death and cause serious financial burdens on humanity. However, the arduous process of developing vaccines and drugs is time-consuming and expensive. It has been estimated that about US$500 million to US$1 billion of capital investment is needed for over 10 years to make a drug or vaccine available to the populace. The problem of winning the war against infectious diseases is complicated by the ability of some the pathogens to mutate and alter their genetic makeup, thereby rendering drugs and vaccines inefficacious (Pronker et al., 2011).

The current situation is alarming and demands action, especially in Africa where most of the countries fall under the developing countries category where a small percentage of annual budgets are earmarked for the healthcare of the citizenry (Yadav and Rawal, 2015a; Yadav and Rawal, 2015b; Yadav et al., 2016).

The present situation requires an aggressive alternative way of combating these medical threats. This may require that Africa look inwards and take advantage of some of her natural bioresources such as plants, mushrooms, and animal products to combat these emerging diseases. The current study aimed at identifying prevailing emerging diseases in Africa, their imposed burden and challenges on the population, enhanced prevailing factors, and the way forward. *Ganoderma lucidum* (Reishi mushroom), a commonly encountered mushroom in Africa was targeted in this review as a potential therapeutic solution to the threats of emerging diseases in Africa by elucidating produced bioactive components in *G. lucidum* and their medical importance. This mushroom which is popularly known as Reishi in Asia or Lingzhi is red-coloured and has remained useful in traditional medicine in Asia, especially China for centuries Chang at al., 1986. It is known as the mushroom of immortality (Cao et al., 2012). In the Pharmacopoeia of the People’s Republic of China, the mushroom is reported to have been used for over two millennia (Wu et al., 2013; Luangharn et al., 2021). Traditional Chinese books classify *Ganoderma* species based on basidiocarp colouration (Szedlay, 2002). Many high and low molecular weight bioactive compounds have been isolated from *Ganoderma* sp. (Ahmadi and Riazipour, 2007; Chan et al., 2007; Chen et al., 2007). Some of these bioactive constituents are; polysaccharides, protein, sterols, and triterpenoids that possess considerable and highly significant therapeutic properties. Others include antibacterial, antifungal, antiviral, immune-boosting ingredients, anticancer, antitumor, antioxidative agents, anti-inflammatory, and anti-hypotensive making *Ganoderma* sp a well-known functional food (De et al., 2012a; De et al., 2012b; Singh et al., 2013; Richter et al., 2015; Hyde et al., 2019; Luangharn et al., 2021). Several studies have revealed *G. lucidum* extract to contain components with an extensive range of pharmacological and therapeutic properties which include immunomodulation, hepatoprotective, hypocholesterolemic, free radical scavenging, and anti-inflammatory (Nahata, 2013; Richter et al., 2015; Luangharn et al., 2021). *G. lucidium* extracts are given as supplements or medicine for several ailments and diseases (Zheng, 2011). Interestingly, the absenteeism of side effects and the huge health aids associated with this mushroom make it ideal and acceptable as herbal medicine (Sanodiya et al., 2009).

## Emerging diseases

### Emerging diseases in Africa

Diseases can be defined as any situation that causes harmful deviation from the normal functional or structural state of the body organ (system), the psyche, or an organism in total. Some of these impairments or damages are generally accompanied by specific signs and symptoms which are significantly different from physical injury. The organs and/or systems’ functional impairment brought by disease could be a result of the intrinsic or extrinsic factors (Nii-Trebi, 2017). Those that emerge from inside the host which might be about the organism’s genetic features are referred to as the intrinsic factors. This may as well be due to any dysfunction inside the host that interferes with the natural processes in a body system. Extrinsic factors refer to those that are external or foreign to the host.

Emerging diseases can be described as an infection newly being discovered inhabitants or are swiftly increasing in occurrence in a geographical range (Morse, 2004). Emerging diseases occur globally irrespective of continent or country and their origin and nature are becoming increasingly difficult to predict due to factors such as extensive international travel, wide and borderless trade policies etc. However, reports on the emerging diseases have revealed that they originate or emerge chiefly from where there is a dense population of diverse animal species, frequently in close interaction with humans (Brown, 2004). New diseases frequently emerge and come into existence while those that have been earlier suppressed or eliminated at times tend to re-emerge as human lifestyles change due to advances in technology, population increase, and changes in social behavior. Around 75% of the emerging diseases affecting people in the past years were documented to originate from animals and/or their products (WHO, 2007).

Out of this 75% documented emerging diseases, an estimated 60% are zoonotic. Some of the most recent are; ebola haemorrhagic fever, severe acute respiratory syndrome (SARS), H1N1, avian influenza, and probably human immunodeficiency virus/acquired immune deficiency syndrome (HIV/AIDS). Several factors have been recognized to be responsible for the spike in the incidence of emerging diseases. Some of these include; overpopulation, expansive international trade, high movement of animal species, climate change, civil unrest/wars, a mutation in microbes, and disruption of the ecology of many animals and insects (Morse, 2004). It is obvious from these factors that the occurrence of emerging diseases will not abate but may likely be on the increase shortly.

### Emerging infectious diseases

In 1987, Joshua Lederberg, Robert B. Shope, and Mary Wilson officially delineated the word emerging and reemerging diseases (E&RD). EIDs are infectious diseases origin whose occurrence in human beings has either surged in less than the last 2 decades or presently threatened to escalate (Oaks et al., 1992). EIDs are those diseases that arise *via* the influx or attack of a host by a foreign body whose actions mar the normal functioning of the host’s systems (Tibayrenc, 2007; Nii-Trebi, 2017; Burrows and Scarpelli, 2020). EID has chiefly been referred to as those infectious diseases that have previously existed or are currently emerging in a population but are escalating very fast in incidence or geographic range (CPC, 2015; WHO, 2015; Fenollar and Mediannikov, 2018) while their origin had been generally linked to and catalyzed by social status, economic state, environmental factors and ecological factors (Fenollar and Mediannikov, 2018).

EIDs are majorly classified into emerging if they are observed in an infected person for the first time and reemerging which have been described as one that re-emerged, historically in a more pathogenic way and swiftly spiral incidence after noticeable control or eradication (Racaniello, 2004; Barber and Stark, 2015). Emerging infections (EIs) appeared exponentially in the track of human history and have engendered limitless damage to the human race (Cohen and Larson, 1996).

Pre-emergence, localized emergence, and pandemic emergence have been documented as the three (3) phases of zoonotic disease emergence (ZDC) (Morse et al., 2012). Additionally, Hughes et al. (2010) reported another five (5) phases of ZDC including, restricted to animals, chiefly human infections, limited human-to-human transmission, continuous human-to-human transmission, and limited to humans.

### The burden of EID in Africa

The costs of emerging infectious diseases (EIDs) are cosmic both concerning lives lost and the economic burden (WHO, 2019a; WHO, 2019b; Bernasconi et al., 2020; Callaway, 2020; Nkengasong and Tessema, 2020; World Bank, 2020). Globally, the world has seen a diverse upsurge of EIDs since the onset of the 21st century to date with acclaimed public health concerns. These include but are not limited to Middle East respiratory syndrome coronavirus (MERS-CoV) for 10 years running, Zika virus (2015–2016), acute respiratory syndrome-related coronavirus (SARS CoV) in 2003–2004, H1N1 swine flu in 2009, and Ebola virus between 2013 and 2016 are few examples (Kaner and Schaack, 2016; Aleanizy et al., 2017; Pereira et al., 2018). Africa had been described to be the home and origin for both emerging and re-emerging infectious diseases with varying degrees of mortality (Dye, 2014; Fenollar and Mediannikov, 2018). Africa’s primary burden arising from endemic diseases is the biggest in the world (Nkengasong and Tessema, 2020). Tuberculosis, Ebola, malaria, measles, along with the lately emerged acute respiratory syndrome coronavirus 2 (SARS-CoV-2) and many others are the tenable examples of emerging infectious diseases that ravaged public health in Africa in recent times (Calvignac-Spencer et al., 2012; Fenollar and Mediannikov, 2018; Nyaruaba et al., 2019; Kalk and Schultz, 2020; Oyejobi et al., 2020). The detailed descriptions of the majority of Africa’s emerging infectious diseases (EIDs) with their origins, causative Agents/Host, countries affected, cases, and transmission in the past decade is shown in Table 1 (Nkengasong and Tessema, 2020; WHO, 2021; Nyaruaba et al., 2022; Phoobane et al., 2022).

**TABLE 1:** Emerging Infectious Diseases in Africa in the past decades.

Disease	Origin	Causative agents/Host	Transmission	Countries affected	References
COVID-19	China	SARS-CoV-2 and Bats	Respiratory droplets and contaminated surfaces and hands	All countries and still Ongoing	WHO (2021)
Zika	Uganda	Zika virus and Mosquitoes spp.	Mosquito bite, sex, mother to foetus and organ transplant	New Guinea, Cape Verde	Nyaruaba et al. (2019); Posen et al. (2016)
Chikungunya fever	Tanzania	CHIKV and Mosquitoes	Infected mosquito bite	Chad, Congo, Sudan, Kenya, Kenya, Somalia, Senegal	Nyaruaba et al. (2019)
Rift Valley Fever	Kenya	RVF virus and Mosquito spp.	Blood or organs of infected animals, raw milk and mosquitoes	Kenya, Gambia, Kenya, Angola, Niger, Uganda, Senegal	Nyaruaba et al. (2019)
Yellow fever	Not determined	Virus and Mosquitoes	Mosquito bite	Senegal, Guinea, Nigeria, Ethiopia, South Sudan, Uganda, Angola, Kenya, Cameroon, Chad, Congo, Ghana, Sierra Leone, Cote d’ Ivoire	Nyaruaba et al. (2019)
Dengue Fever	Not determined	Dengue virus and Mosquitos	Mosquito bite and mother-to-child	Burkina Faso, Cote d’ Ivoire, Burkina Faso	Nyaruaba et al. (2019)
Ebola Virus Disease	DRC	Ebola virus and Bats or NHP	Infected animals, person-to person and *via* semen	Guinea, Uganda), MAli, Liberia, Guinea, Nigeria, Sierra Leone, DRC, Senegal, Uganda, Uganda	Petersen et al. (2018); Rohan and McKay (2020)
Measles	Not determined	Measles virus and Humans	Infected person coughing or sneezing	Burundi, Tunisia	Furuse et al. (2010)
Monkeypox	DRC	Monkeypox virus and Unknown natural host	Human-to-human, wild animals	DRC, Nigeria, Cameroon and Nigeria and Central African Republic (CAR)	Beer and Rao, (2019)
cVDPV2	Not determined	Reverted live attenuated OPV and humans	Person-to-person	Sudan, Somalia and Nigeria	Jorba et al. (2016)
Poliomyelitis	Not determined	Poliovirus and Humans	Person-to-person (fecal oral route)	Madagascar, South Sudan, Madagascar, Cameroon, Equatorial Guinea, Cameroon, Chad and Nigeria	Mehndiratta et al. (2014); Jorba et al. (2016)
Lassa Fever	Nigeria	Lassa virus and *Mastomys* rats	Exposure infected *Mastomys* rats’ urine or faeces and person-to-person	Nigeria, Liberia, Benin), Togo and Ghana	CDCP (2019)
Marburg Hemorrhagic Fever	Germany	Marburg virus, African fruit bat and *Rousettus aegyptiacus*	Fruit bats and human to-human	Uganda	CDCP (2021b)
Bird flu	China	Influenza virus (H5N1) and Birds	Contact with infected poultry	South Africa and Egypt	Kayali et al. (2014)

In addition, factors such as massive international travel and trade, demographic change, and climate change are drivers of EIDs in Africa (Fenollar and Mediannikov, 2018; Nyaruaba et al., 2019). Hence, when it comes to readiness to manage EIDs, African countries can be said to be ill-prepared in comparison with other continents (Braack et al., 2018; Fenollar and Mediannikov, 2018; Petersen et al., 2018). Factors such as political upheaval, economic quagmire, and massive rural-urban migration are contributing factors to the breakdown of many health systems that lack appropriate disease surveillance that will assist in managing, predicting, and preventing the impending EIDs in Africa (Shears, 2000). Furthermore, poverty, poor healthcare, and total reliance on donor countries to finance scientific studies related to EIDs aggravate health problems and at the same time reduce the effectiveness of Africa’s progress in combatting EIDs (Shears, 2000; Fenollar and Mediannikov, 2018). African countries are grouped amongst the least concerning budgetary provisions for health and accessibility to health personnel. In addition, Africa is the leading continent for infant mortality with five of the six worst countries in the world being on the continent. Currently, the continent loses 100 of every 1,000 births as reported by World Health Organization (Fenollar and Mediannikov, 2018). Fifty percent (50%) of the deaths in Africa are caused by infectious diseases compared to just two (2) percent in Europe, underlining the need for Africa to take the lead in the war against infectious diseases.

## Causes and transmission of infectious diseases in Africa

### Major causes

The key factors of infectious disease emergence include microbial, human, and environment. However, the complex interaction of these factors determines the potential virulence of the pathogens (Marty et al., 2001). The emerging and re-emerging diseases come about and reappear over and over again. Infectious disease agents (bacterial, fungi, protozoa, helminths, and viruses) undergo different phases of adaptation to build up or gain diverse pathogenic features or potentials in a new host before causing an epidemic (Carruthers et al., 2007). Microbial agents are developed by processes such as natural, controlled, or uncontrolled gene mutation and genetic recombination along with other factors which make the infectious agents develop and acclimatize to new vectors or hosts’ ecological niches and spread quickly (Alcais et al., 2009). Several factors have been established to give rise to this adaptation and subsequent emerging disease. Howbeit, the multifaceted association sandwiched between the infectious agents, the hosts, and the environment is vital.

The environment is affected by deforestation and expansive agricultural development and upgrading which cause potential alterations in the ecology of microbes and intensify their adaptation to human host (Neiderud, 2015; Tong et al., 2015). Furthermore, sociodemographic factors including a rise in population density, drop in standards of living, dearth of infrastructure, human traveling, dispute, and social unpredictability as well as the killings of wild animals for meat brought about the rise in host-microbe contact that promotes infections in humans (Utzinger and Keiser, 2006; Gayer et al., 2007; Defo, 2014). Also, certain conscious human activities such as the use of biological weapons in warfare have contributed to the incidences of infectious diseases. Moreover, mutation or changes in the genetic makeup of a pathogen, which can occur due to contact with chemical reagents and antimicrobial agents (like an antibiotic), might result in gene impairment and emanation of drug-resistant pathogen variants or strains that may perhaps give rise to new disease (Lashley, 2004; Alcais et al., 2009).

Predominantly, EIDs caused by viral pathogens are accountable for a large percentage of emerging infectious diseases, two-thirds of the infectious diseases documented are caused by viruses (e.g., Filoviruses, Ebola, and Marburg) (Dye, 2014; Brown et al., 2018; Lado et al., 2019). Few of these infectious disease pathogen emergences and causes are discussed in Table 1.

### Transmissions

Transmission of infectious diseases is mostly brought about by contact with an infectious agent such as bacteria, viruses, fungi, protozoa, helminths, etc., or sometimes with an infected person (Barber and Stark, 2015; Pieterse, 2019). However, several factors like global urbanization, population density increase, social upheaval, traveling, agricultural practices, and climatic change coupled with some other human actions and events that decimate microbial environment have been acknowledged as an avenue for infectious agents to gain entrance into human hosts (Pavia, 2007). The more they increase in human engagement exposes them to reservoirs of disease-causing organisms, the more the risk of contracting new diseases. It has been affirmed that most of these pathogens are transmitted through an intermediate animal host, mostly rodents which now get in contact with humans due to environmental and behavioral factors (Table 1) (WHO, 2001; Morse et al., 2012).

Nanyingi et al. (2015) attributed the large West African RVF occurrence in humans and animals in 1987 to changes in the environmental conditions and the actions between animals and humans as the major factor behind the outbreak. Pathogens may be contracted *via* urine and droppings of the animals which might be aerosolized and infect vertebrates as well as man. For instance, Hantavirus Pulmonary Syndrome, Lassa fever, and the Nipah virus encephalitis (Table 1), in which pathogenic viruses coexist with specific rodent species (Tadin et al., 2016). Few of these infectious disease pathogen emergence and transmission in humans are reported in Table 1.

## Emerging diseases in Africa: The challenges and the way forward

### The challenges

Regardless of outstanding improvement in the past (especially past 2 decades), the continuous mortality arising from incidences of emerging infectious diseases are still been reported (Nii-Trebi, 2017). The difficulty in dealing with these diseases, especially for accurate time prediction, and location/uniqueness of the causative pathogen is real and presents a major challenge in combating them (Oyston and Robinson, 2012; Mukherjee, 2017; Wong and Qiu, 2018).

Some basic concept in infectious disease emergence has been described by Wilson, (1995). These include the complexity of the infectious disease’s emergence, the dynamism of the infectious diseases, agents involved in new and re-emerging infections cross taxonomy (such as viruses, bacteria, fungi, protozoa, and helminths), incorrect diagnosis of the pathogen, human activities, disease patterns and emergence which are sharpened and influenced by social, economic, political, climactic, technologic and environmental features coupled with the current global situation which favours disease emergence.

Genetic modification and changes that occur in pathogenic microorganisms have remained one of the challenges associated with emerging diseases. The propensity of some of the pathogens to genetically modify on their own has presented researchers and healthcare experts with serious headaches in dealing with them. This is due to the emergence of new phenotypic and genotypic features which most of the time enhance the virulence or resistance of the pathogen to vaccines and drugs. Sometimes, they improve the ability of the pathogen to colonize a wider range of hosts or reduce the time of their incubation. This, therefore, encourages the emergence and re-emergence of infectious diseases, triggering new epidemics repeatedly. Several illustrations of emerging and re-emerging infectious agents with the capacity to undertake manifold genetic modifications and advance in retort to altering the host and environmental conditions have been documented (Tibayrenc, 2007). The emergence of EIDs menace thus persists since the pathogens endure to undergo genetic modification while the anthropological and environmental conditions that encourage pathogen adaptation to infection in humans remain. Nevertheless, findings had suggested the complex nature of various factors other than the only genetic variation that may lead to the virulence of pathogenic microorganisms resulting in the occurrence of infectious diseases and the severity of such infections (Fineberg and Wilson, 2010).

Another major challenge emanating from genomic modification is the incidence of resistance to drugs, especially antimicrobial resistance (De Clercq, 2009; Meyer et al., 2011; Fauci and Morens, 2012; Klein, 2013). There has been much concern about the increased cases of drug-resistant pathogens in recent times. Some of the cases of resistance may not be due to genetic modification but to drug abuse by the patients. In Africa, some patients are in the habit of discontinuing their drugs immediately after they feel better despite the presence of the pathogen in their systems. This makes the pathogen in some cases build resistance to some of the antibiotics so abused. Some of the organisms found to have developed such resistance include but are not limited to *Staphylococcus aureus*, *Escherichia coli*, *Neisseria gonorrhoeae,* etc. (Lemon et al., 2007).

### The way forward

Pathogens responsible for causing Infectious diseases have somehow perfected their ability to appear and also escalate speedily by any imaginable means, exhibiting a higher pathogenic potential and mutation rate to resist drug attacks. Sequel to the widespread of pathogens, some of which are not yet recognized, and the wide range of animal species involved as vectors or hosts, a lot still needs to be done in combating infectious diseases and improving public health. Hence, to address the global challenges of EIDs and associated social and economic risk, there is a need for the development of an effective strategy for the deployment of drugs and vaccines when necessary. There is also a need for training and retraining of skilled personnel for comprehensive research, prompt diagnosis, management, and treatment of patients. The building of infrastructures and capacity for state-of-the-art equipment and technologies for diagnosis, screening, *in vitro* trials, and treatment cannot be overemphasized. Attention must also be given to international cooperation in research and active national, continental and global networks for severe infectious diseases along with improving the biological materials to boost antimicrobial product advancement and vaccine trials.

There is a need for distinct observation on conditions that encourage disease emergence, specifically those of human events that destroy the environment and change ecological settings thereby increasing animal interaction with humans. As long as this is not stopped, there is, then, no evidence that advancement in the detection and control of infectious disease plans will adequately prevent novel diseases from appearing since each novel disease comes with peculiar problems. Moreover, there must be a breakthrough in predicting zoonotic emerging diseases to further boost the rate of success in dealing with these infections. To meet the unique challenges posed by EIDs, an innovative and efficient formulation and utilization of the local medicinal plants and macrofungi products could be a promising alternative approach. The medicinal importance of copious local medicinal plant products such as *Ganoderma lucidum* (GL) has been documented by several researchers and the possibility of GL bioactive compound being a novel solution to EIDs in Africa is reported in this review.

## Ganoderma lucidum

### History and taxonomy of *G. lucidum*

GL was presented by Curtis in 1881 (Karsten, 1881) garrison on material belonging to Peckham, London, United Kingdom, and the identification was approved by Fries in 1821 (Chen, 1999). GL has formerly been treated as identified as *Boletus lucidus,* Curtis (1777) (Fr.), *Polyporus lucidus* Curtis Fr (1821) *Polyporus polychromus* Curtis (Fr.), *G. polychromum* Curtis (Fr.), *G. sessile* Curtis (Fr.) and *Fomes lucidus* (Curtis) Sacc. (Siwulski et al., 2015). GL was reported in China for the first time by Sydow et al. (1907) while Teng (1934) delineate the assemblage of GL from various regions in China (Wang et al., 2012). The monograph of traditional Chinese medicinal fungi coupled with the account of GL as Lingzhi was compiled by Liu (1974) in his book (Hapuarachchi et al., 2015). Ever since, GL has been scientifically acknowledged as the same as Lingzhi in different research papers and reports on Chinese edible and medicinal mushrooms (Ying, 1987; Mao 1998; Dai et al., 2017). For about two thousand years, GL*,* a woody polypore (Basidiomycota) has been generally utilized as a medicinal mushroom or supplement in China (Sliva, 2006) and has remained a significant part of the traditional medicine ingredients in the far east, particularly in China and Japan. GL has been broadly employed in the branding of commercialized Lingzhi products in the global mushroom industry due to its several medicinal benefits (Lai et al., 2004). Taxonomically. GL belongs to the Kingdom fungi which include heterotrophic nonvascular organisms that have a chitinous cell wall. Spore production (sexual/asexual) is the means of their reproduction and grows only through means of vegetative hyphae. They belong to Basidiomycota Phylum, Agaricomycetes class, Polyporales order, and Ganodermataceae family.

**FIGURE 1:**
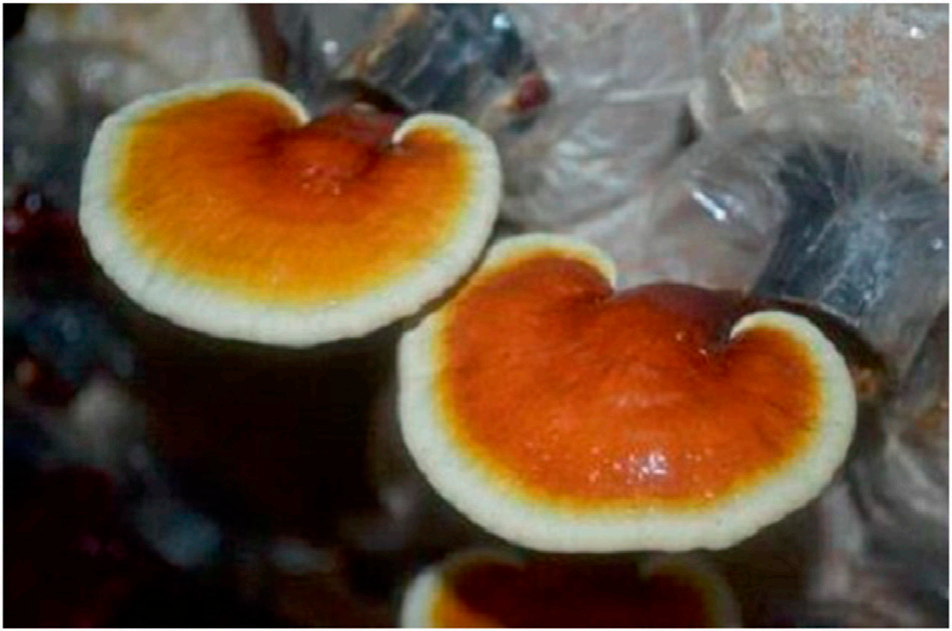
Image of *Ganoderma lucidum*.

They grow mainly on wood as decayers and can be as well as be parasites or symbiotics, but they are terrestrial fungi. The species of GL has a defining characteristic that separates it from other species in the genus *Ganoderma* in that an association with hardwood trees is necessary. In addition to this distinct characteristic, the hyphal walls of the GL are thicker than other species in the same genus (Figure 1).

GL is a commonly known therapeutic mushroom that has remained useful for the prevention and treatment of different disorders to enhance longevity and health mostly in China, Japan, and Korea. It was known as *God’s herb* in ancient China as it was affirmed to extend life, boost the youthful spirit and sustain/preserve liveliness.

## *Ganoderma lucidum* distribution and cultivation

### Distribution

GL has a global distribution but mainly grows and is found in subtropical and moderate weather regions, particularly in the Asia (China, Korea, and Japan), Europe (Sweden, Denmark, and Poland), Africa (Kenya, Tanzania, and Ghana) and America (North and South America) (Pilotti, 2005; Cao et al., 2012; Wang et al., 2012; Cao and Yuan, 2013; Siwulski et al., 2015). GL grows in the areas around Yangtze and Yellow rivers in China and its range extends as far north as Canada and south into Argentina (Tiqiang and Kaiben, 2004). The basidiomata of the genus Ganoderma are bound and described as sessile to stipitate, with double-walled basidiospores and internal pillars (Karsten, 1881; Moncalvo and Ryvarden, 1997). According to Kirk et al. (2008), 80 species of *Ganoderma* are recognized globally, while two global fungal databases viz. Index Fungorum (Index Fungorum, 2021) and MycoBank (MycoBank, 2021) hold 459 and 503 records, respectively.

### Cultivation

**FIGURE 2:**
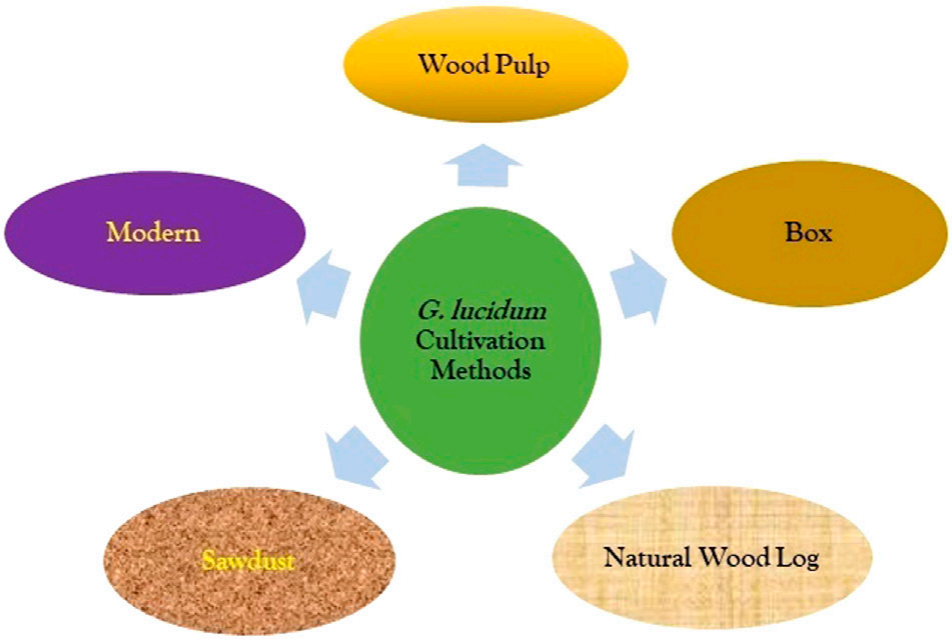
Different methods of *G. lucidum* cultivation.

The high demand for GL due to its uses in several industries like food, therapeutic, cosmetics, and other health-related uses has increased the rate of cultivation of GL among other mushrooms (Yang et al., 2019). Several approaches have been used to cultivate the mushroom and they have all proven effective (Renu and Brij, 2015). Three main methods utilized for the cultivation of GL are the natural wood log cultivation method (most popular to date), wood pulp cultivation (Bottle cultivation), box cultivation, and cultivation of sawdust and modern cultivation practices (Figure 2), which are still in use to date. In the wood log cultivation method, hardwood logs/chips are left in a warm, moist environment and are always used for growth, then the moistened wood log is inoculated with spores for germination/sprout. This can be carried out both in the lab and in relaxed settings while the whole growth cycle can take up to 1 year. Cilerdzic et al. (2018) reported the growth of the GL fruiting bodies on hardwood logs, stumps, and sawdust. Cultivation of GL can also be done using wood chips and chemicals in vacuum packing. However, this yields a much faster harvest in good time (around 2 months) but produces an inferior quality product. The artificial cultivation of GL had been documented to take some time and the quality is based on the environmental conditions. However, mycelium production has been observed in both liquid and solid-state fermentation while GL secondary metabolites can be acquired rapidly by fermentation technology (Zhou et al., 2014; Yang et al., 2019).

The existence of *G. lucidum* in different parts of Africa has been reported. Different methods of cultivating *G lucidum* have been successfully established *via* several studies in different locations within Africa. Diverse substrate formulations have been reported and employed for the large-scale production of this mushroom to meet its demand. In Lagos Nigeria, Adongbede and Atoyebi, (2021) cultivated *G. lucidum* (Curtis) P. Karst. on the sawdust (substrate) of six indigenous hardwoods supplemented with rice and wheat bran. A higher yield of *G. lucidum* was obtained with these local materials compared to the report of Thakur and Sharma (2015) when cultivated on sawdust supplemented with wheat bran in India. Also, Ihayere et al. (2017) reported impressive results on the cultivation of ten (10) strains of *G. lucidum* from different locations in the tropical rainforest zone of southern Nigeria with isolates from *Iguikhinwin (Edo State) and Oghara (Delta State)* showed better *mycelium biomass production*.

### Bioactive component of *G. lucidum*

GL bioactive constituents have recently gained remarkable recognition. Countless bioactive components have been reportedly isolated from the fruiting body, mycelia, and spores of GL (Table 2). About 400 different bioactive compounds from GL have been documented. The major identified active compounds possessing biological activities include polysaccharides, triterpenoids, nucleotides, sterols, amino and fatty acids, meroterpenoids, sesquiterpenoids, steroids, alkaloids, polysaccharides, volatile oils, proteins, and many more (Yang et al., 2019; Vo et al., 2021). Some of these key bioactive constituents are shown in Table 2. Nevertheless, GL bioactive constituents are dependent on different conditions including the origin, classifications, cultivation process, method of extraction, etc (Vo et al., 2021). GL polysaccharides and triterpenoids are considered the most bioactive compounds with various health advantages and have increased request in the market (Vo et al., 2021). The report from the Chinese Pharmacopoeia (2015 edition), showed a minimum of 0.90% polysaccharides and 0.50% triterpenoids are contained in the *Ganoderma* dry fruiting body (CPC, 2015; Vo et al., 2021). Nevertheless, triterpenoids and polysaccharides have attracted significant and worthwhile attention due to their diverse importance and high content in fungus. Some of these bioactive compounds are reviewed below:

**TABLE 2:** Common pharmacological effects of *G. lucidum* main bioactive compounds.

Pharmacological effects	Main bioactive compounds	References
Anticancer	Polysaccharides (1→3, 1→4, and 1→6-linked β and α-D (or L)-glucans)	Wachtel-Galor et al (2011); Ferreira et al. (2015)
	Glycopeptides and peptidoglycans	Ferreira et al. (2015); Cör et al. (2018); Hapuarachchi et al. (2015); Sudheer et al. (2019)
	Triterpenoids (Ganoderic acids, ganodermic, ganolucidic acids, ganoderals, ganoderiols, lucidumol, lucialdehyde, lucidenic acids)	Boh (2013); Duru and Çayan (2015)
Immunomodulatory	Protein Ling Zhi-8 (LZ-8), lectin, ribosome inactivating proteins, glycopeptides/glycoproteins, peptidoglycans/proteoglycans, ganodermin A, ribonucleases, proteinases, metalloproteases, laccases	Cao et al. (2012); Sudheer et al. (2019)
Antidiabetic	Polysaccharides, proteoglycans, proteins (LZ-8) and triterpenoids	Ma et al. (2015); Ahmad, (2020); Ma et al. (2015)
Anti-inflammatory	Ganoderic acids T-Q and lucideinic acids A, D2, E2, and P	Sliva et al (2003); El Mansy, (2019)
Antioxidant	Triterpenes, polysaccharides, polysaccharide peptide complex and phenolic component; Methanolic extracts; Phenolic and polysaccharide extracts	Kan et al (2015); Yildiz et al (2015); Kumari et al. (2016); El Mansy, (2019)
Cardiovascular problems	Polysaccharides (Ganopoly)	Gao et al. (2004)
Antiviral	Triterpenoids against Enterovirus 71; Ganoderic acid derivatives against H5N1 and H1N1 influenza; Ganoderiol F, ganodermanontriol against HIV-1	Zhu et al. (2015); Bishop et al. (2015); Zhang et al. (2014); Zhu et al. (2015)
Antimicrobial	Polysaccharides; Triterpenoids (ganoderic acids, ganodermin, ganoderic acid A, ganodermadiol, ganodermanondiol, lucidumol B, ganodermanontriol, ganoderic acid B, ganolucidic acid B)	Cör et al. (2018); Hapuarachchi et al. (2017); Cör et al. (2018); Sudheer et al. (2019)
	Aqueous and methanolic extracts; Triterpenes, ganomycein, and other aqueous extracts	Sudheer et al. (2019); Stojkovicć et al (2014); Hleba et al (2014)
Sterols	Provitamin D2	Wachtel-Galor et al (2011)

### Triterpenoids

More than two hundred (200) triterpenoids were confirmed in *G. lucidum’s* fruiting bodies, spores, and mycelia (Xia et al., 2014; Baby et al., 2015). Generally, these obtained triterpenoids had been classified into two groups, the carboxylic side chain (*Ganoderma* acids) group and the other group with no carboxylic side chain (*Ganoderma* alcohols). A huge chunk of them is lanostane type triterpenes while some are regarded as lucidenic acids (Weng et al., 2007; Xia et al., 2014). Ganoderma Triterpenes are further grouped as ganoderic acid, ganoderiol, ganoderone, ganolactone, and ganoderal, according to their functional groups and side chains (Xia et al., 2014; Baby et al., 2015). The existence of these triterpenoids, particularly ganoderic acid, the major triterpenoid in this mushroom is responsible for the bitter taste in *G. lucidum* (Sudheer et al., 2019). Ganoderic acids are of different types which include, GA-A, B, C, and F. Research has shown a higher significant presence of triterpenoids in the spores in comparison to other parts of the mushroom (Yu et al., 2016; Sudheer et al., 2019). Furthermore, the production of bioactive compounds in *Ganoderma* is influenced by the zone and under which conditions they are cultivated while the structure of lanosterol determines the basic structure of triterpenoid (Min et al., 2001). However, according to the number of carbon atoms and functional groups, triterpenes are grouped into three groups of which many of which have been documented to be useful as chemotherapeutic agents (Luo and Lin, 2002).

### Polysaccharides and peptidoglycans

Reports have shown the presence of polysaccharides in GL*.* Over 100 different kinds of polysaccharides observed in GL have been documented (Sudheer et al., 2019). Several studies have revealed GL as a very good source of polysaccharides and glycopeptides (Nie et al., 2013). Polysaccharide is a compound formed from many monosaccharides, connected by glycosidic bonds while most polysaccharide belongs to a group of β-glucan which involves a linear backbone of β- (1,3) and connected by D-glucopyranosyl groups with various degree of branching from the C-6 position (Sudheer et al., 2019). Several components, structures, molecular weights, and effects of GL polysaccharides are noticeable at different growth phases of GL. The highest content of polysaccharides was contained in the mycelium while the lowest content was reported in the fruiting body.

Furthermore, glucose and galactose are the major sugars in the fruiting bodies while glucose was reported as the main monosaccharides from the mycelium and spores (Khanna et al., 2012). Countless types of polysaccharides having a range of molecular weights from 4 × 105 to 1 × 106 Da present in the fruiting body and mycelia of GL have been documented (Khanna et al., 2012; Bishop et al., 2015; Ferreira et al., 2015). However, the rudimentary framework of GL polysaccharides (GLPs) is made up of a high-molecular-mass β-(1→3)-d-glucan coupled with (1→6)-β-d-glucosyl branches, and the key sugar components remain mannose, rhamnose, glucose, and galactose (Liu et al., 2014). β- Glucans of higher molecular weights have been revealed to be more efficacious than glucans that have low molecular mass (Gao et al., 2002; Chang and Lu, 2004; Huie and Di, 2004). The presence of chitosan in GL has worthwhile features in specialized sectors including medicine, pharmacy, and cosmetics (Mesa et al., 2015). GL has been shown to contain proteoglycan which has antiviral activity as a result of peptidoglycan’s presence (Ji et al., 2007).

### Steroids and ergosterol

Steroids of over 20 kinds have been reportedly obtained in GL and their structures can be classified into ergosterols and cholesterols (Baby et al., 2015). Sterols are derivatives of triterpenoids, however, the presence of ergosterol and 24-methylcholesta-7,22-trien-3-ol coupled with 8,9-epoxyergosta-5,22-dien-3,15-diol has been reported in GL as the first isolated free sterol (Huie and Di, 2004). Hajjaj et al. (2005) reported the isolation, purification, and identification of 26-oxygenosterols. Zhang et al. (2008) documented a new, highly oxygenated sterol, 22E, 24R-ergosta-7,22-diene-3beta, 5alpha, 6beta, 9alpha and 14alpha-pentol. Ergosterol compound is a vitamin D precursor and remains essential and pharmaceutically relevant. The integrity of the fungal cell membrane is said to be preserved by ergosterol which also produces cellular energy and its measurement remains the key parameter in biomass production. Lv et al. (2012) documented higher ergosterol in GL compared to other species of *Ganoderma*.

### Proteins and polypeptide

Biologically active proteins of various types from GL have been identified. *G. lucidum* has been documented as a natural source of proteins and peptides with biological properties (Xu et al., 2011). Lin et al. (2011) reported Lin Zhi-8 (LZ-8) which is a polypeptide that consists of 110 amino acid residues with a molecular mass of 12 kDa coupled with an acetylated amino terminus. The sequence and projected secondary structure of LZ-8 are homogenous to the variable region of the heavy chain of immunoglobulins (Yang et al., 2019). LZ-8 remains the primarily first immunomodulatory protein isolated from the mycelial extract of GL by employing chromatographic and electrophoretic techniques in 1989 (Ahmad, 2018; Hsu and Cheng, 2018). However, their mode of action is almost identical to lectins which have the mitogenic capacity (Kawagishi et al., 1997) towards mouse spleen cells and human peripheral lymphocytes *in vitro*. Ribosome inactivating proteins (RIP), antimicrobial proteins, ribonucleases, and laccases are some of the other proteins, and all play a vital function in regulating the human body’s immune system directly or indirectly. Furthermore, ganodermin with 15 kDa molecular mass is another protein isolated from the GL fruiting bodies and has antifungal activity.

### Lipids and fatty acids

Phosphatidic acids are one of the lipids reported in GL. However, phosphatidic acids do not have a significant presence (quantity-wise) in living organisms, but are very important in the transportation of materials across the membrane and protecting the body against damage and infection during inflammation (Hsu and Cheng, 2018). Sequel to these lipids, GL is considered important among medicinal mushroom species (Gao et al., 2003). On the other hand, palmitic acid, linoleic acid, oleic acid, and stearic acid are already identified as the main fatty acids present in GL. Fatty acids obtained from the spores inhibit tumor cell proliferation (Lv et al., 2012). Another fatty acid is nonadecanoic acid with the highest inhibitory property followed by heptadecanoic acid, while palmitic acid and stearic acid remain the strong apoptotic agents (Hardy et al., 2003; Fukuzawa et al., 2008).

### Enzymes

Enzymes such as β-N-Acetylhexosaminidase, α-1,2-mannosidase, endo-β- 1,3-glucanase, β-1,3-glucanase and glutamic protease have been obtained from GL while glutamic protease remains the main protein in the GL extracts (Kumakura et al., 2019; Yang et al., 2019).

### Nucleosides and Nucleotides

Nucleosides and Nucleotides are nitrogenous compounds that perform vital roles in metabolism and stimulate hemopoiesis. The presence of nucleosides such as uridine, adenosine, cytidine, inosine, guanosine, and thymidine, along with nucleotides like uracil, adenine, hypoxanthine, guanine, and thymine has been reported in GL (Gao et al., 2007). Nucleosides include adenosine and 5-deoxy-50 methyl sulfinyl adenosine however, adenosine from GL has been revealed to suppress platelet aggregation and prevent cardiac arrests and thrombosis (Shimizu et al., 1985).

### Amino acids

Only two of the twenty known amino acids are not present in GL, while the most copious amino acid was found to be leucine with a robust antidiabetic and antioxidant activity (Zhang H. et al., 2018; Zhang K. et al., 2018). In addition, the presence of 16 amino acids had been revealed according to the nutritional analysis of GL (Table 3), where glutamic acid (Huang and Ning, 2010), aspartic acid (Liu et al., 2011), glycine, and alanine convey the highest relative abundance of 120, 117, 108 and 100 respectively while methionine displays the least relative abundance of 6 (Wang et al., 2002).

**TABLE 3:** Amino acid composition in G. *lucidum* (Sudheer et al., 2019).

Amino acid	Relative abundance
Glutamic acid	120
Aspartic acid	117
*Glycine*	108
Alanine	100
Threonine	66
Valine	61
Proline	60
Leucine	55
Serine	54
Isoleucine	36
Phenylalanine	28
Arginine	22
Lysine	21
Tyrosine	16
Histidine	12
Methionine	6

### Vitamins and minerals

Vitamins of different types from GL have been documented. Some of these vitamins are C, D, E, B1, B2, B6, and β-carotene. In addition, minerals elements such as iron, sodium, carbon, zinc, magnesium, arsenic, calcium, potassium, phosphorus, chromium, and many others have been identified in GL (Ahmad 2018; Yang et al., 2019).

### Phenolic compounds

Phenolic compounds remain one of the bioactive components contained in GL and are classified as phenolic acids and polyphenols. The phenolic acids include benzoic acid, gallic acid, chlorogenic acid, and syringic acid while polyphenols include stilbenes, flavonoids, and tannins. Researchers have confirmed the presence of these compounds in GL along with their medicinal importance (Mau et al., 2002; Heleno et al., 2012; Kumari et al., 2016; Sudheer et al., 2019).

### Alkaloids and other compounds

In general, the relatively low content of alkaloids in GL had been reported (Sudheer et al., 2019). Huie and Di, (2004) isolated choline and betaine from GL spores (Huie and Di, 2004). Some researchers have identified the existence of alkaloids and their chemical Allies’ derivatives in GL (Ihayere et al., 2010). Mizuno, 1995 reported that GLextracts (% dry weight) comprises folic (68.9%), protein (7.3%), glucose (11.1%), and metals (Chen, 1999; Stamets, 2000). Other compounds like oleic acid, soluble proteins, and cyclo-octasulfur which is an ergosterol peroxide and cerebrosides have been detailed in GL (Gao et al., 2004; Mckenna et al., 2012; Sudheer et al., 2019). Liu, (1999) revealed the presence of stearic acid, choline, palmitic acid, tetracosane, ergosta-22-dien-3-ol, nonadecanoic acid, behenic acid, hentriacontane, ergosterol, betaine, tetracosanoic acid, and β-sitosterol isolated from the spores of GL. Chiu et al. (2000) discovered germanium, with a concentration of 489 μg/g, the fifth-highest among the minerals identified in GL fruiting bodies obtained from the wild (Chiu et al., 2000). Although germanium is not a vital element at low doses, however, it has been established to possess antitumor, antioxidant, antimutagenic, and immunopotentiating, activities (Kolesnikova et al., 1997).

### Nutritional profile of *Ganoderma lucidum*

Globally, for decades, mushrooms have been esteemed as food and medicine (El Sheikha and Hu, 2018). Kaul (2002) reported medicines and food with a common origin/source. Nonetheless, mushrooms remain a substantial unexploited resource in producing efficacious pharmaceutical products, nutrients, and cosmetics. As a matter of fact, according to Fungorum and species, (2020), out of the estimated two to four million fungal species globally, about 150,224 species have been reported to be mushrooms and about 3,000 of those are edible (Hawksworth, 2017). Based on the nutritionist’s perspective, the fresh mushrooms were rich in soluble fibers mainly β-glucan polysaccharides and chitosans, and insoluble fibers (Sadler, 2003). Roy et al. (2015) documented the nutritive value and mineral contents of GL*,* and this is presented in Table 4. An appreciable amount of crude protein and carbohydrates found in GL was reported by Zhou et al. (2007).

**TABLE 4:** Nutritional profile, mineral and vitamin constituents of *Ganoderma lucidum* (El Sheikha, 2022).

Constituents (%)	Content		Dietary recommended intakes for adults (DRIs)	Value in 100 g Mushroom/DRIs *×* 100
	g/100 g Mushroom (wet weight basis)	g/100 g Mushroom (dry weight basis)		
Moisture	47			
Total Solids		53		
pH value				
Energy (kcal)			Men (2,215)	Men (10.79)
			Women (2025)	Women (11.80)
Water-soluble proteins	19.5	36.80	Men (56)	Men (34.82)
			Women (46)	Women (42.39)
Total lipids	3.00	5.66	44–77	3.90–6.82
Total ash		6.3		
Reducing sugars	4.39	8.28		
Nonreducing sugars	1.02	1.92		
Total sugars	5.41	10.21	130	4.16
Crude fibers		3.5	Men (38)	Men (9.21)
			Women (25)	Women (14.00)
Polyphenols (as gallic acid)	0.04	0.08	1	7.5

Minerals	Mineral content (mg/100 g mushroom)	DRIs (mg/day)	Value in 100 g Mushroom/DRIs *×* 100
Potassium	432	4,700	9.19
Phosphorus	225	700	32.14
Sulfur	129	200–1,500	8.60–64.50
Magnesium	7.95	Men (400)	2.00
		Women (310)	2.60
Sodium	2.82	1,500	0.20
Calcium	1.88	1,000	0.20
Copper	26	0.9	2,889
Manganese	22	Men (2.3)	956.52
		Women (1.8)	1,222.22
Iron	2.22	Men (8)	27.75
		Women (1.8)	12.33
Zinc	0.7	Men (11)	6.40
		Women (8)	8.75
Vitamin			
Thiamine (B1)	3.49	Men (1.2)	290.83
		Women (1.1)	317.27
Riboflavin (B20	17.10	Men (1.3)	1,315.38
		Women (1.1)	1,554.54
Niacin (B3)	61.9	Men (16)	386.87
		Women (14)	442.14
Pyriodoxine (B6)	0.71	Men (1.4)	50.71
		Women (1.2)	59.16
Ascorbic acid	32.2	Men (90)	35.77
		Women (73)	42.93

Concerning the nutritional profile of GL*,* it can be inferred that GL possesses a considerable nutritional potential that should stimulate the interest of researchers in different fields of study like pharmaceuticals, nutraceuticals, nutrition, and cosmetics (Chang and Wasser, 2017).

### Therapeutical importance of *Ganoderm lucidum*

*GL* is recognized for its medicinal properties rather than its nutritional value. It is not an edible mushroom because it is a polyphore. It is seen as a mushroom that has a good effect on health and therefore gained the interest of many researchers by studying its bioactive constituents. Several documented works have attested to its antimicrobial, antiviral, and anti-inflammatory effects against many microbial, viral and inflammatory agents. *Ganoderma* is rich in different bioactive compounds with therapeutic effects and they include polysaccharides, triterpenoids lipids, lysosomes, proteins, and nucleotides. It contains certain elements such as germanium, calcium, potassium, calcium, and compounds like alkaloids, flavonoids, and coumarins (Ko et al., 2008).

**FIGURE 3:**
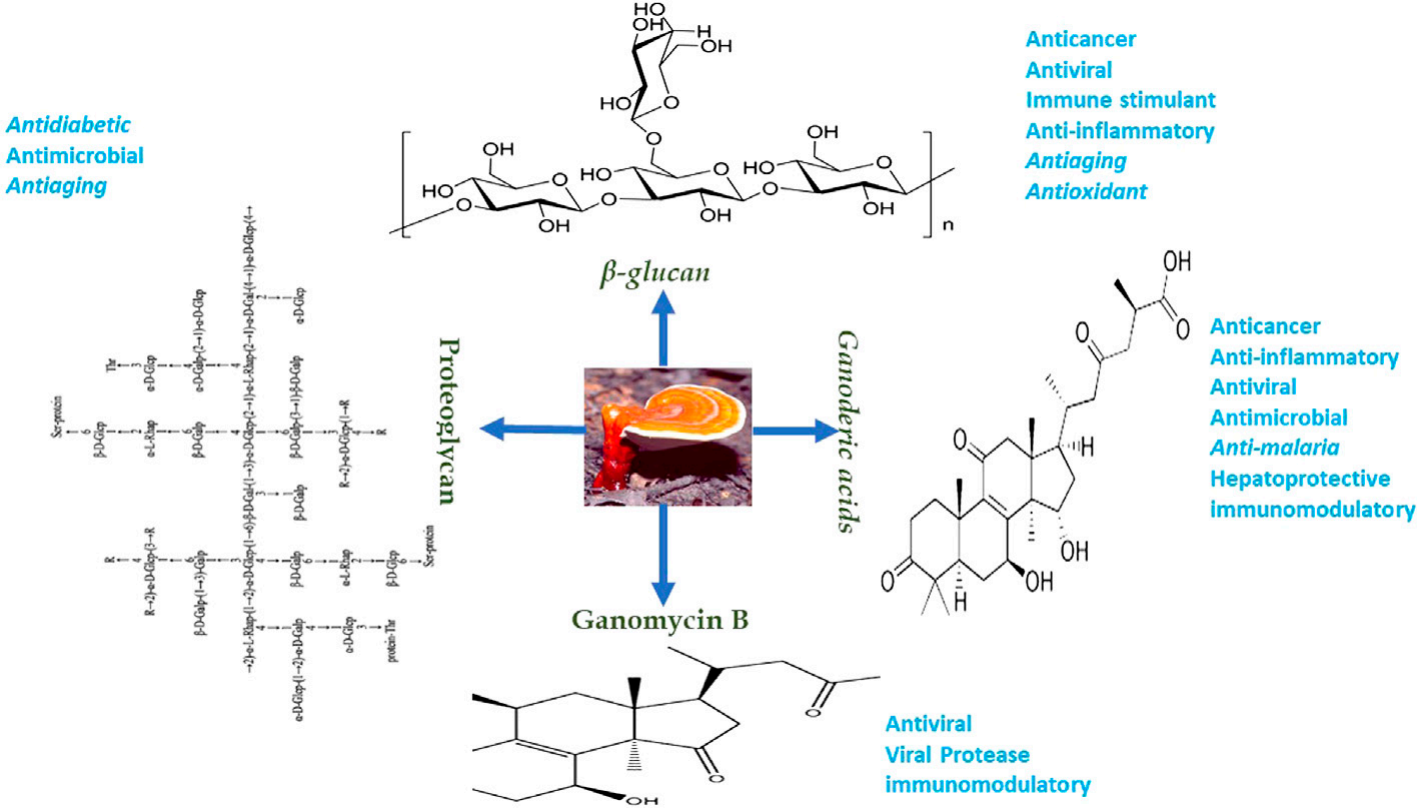
Therapeutic importance of *G. lucidum* bioactive compounds.

Diverse medicinal benefits of GL have been established, and they include anti-tumor (Kao et al., 2016), anti-malaria (Kao et al., 2013; Chen, 2020), anti-microbial (Batra et al., 2013; Cör et al., 2018), anti-inflammatory (Hasnat et al., 2015), and anti-viral effects (Zhu et al., 2015) (Figure 3). Given the commercial and pharmacological potential of GA, biosynthesis of GA by cell factories received serious attention some few years ago (Shi et al., 2013).

### Immunomodulators/immune booster

**FIGURE 4:**
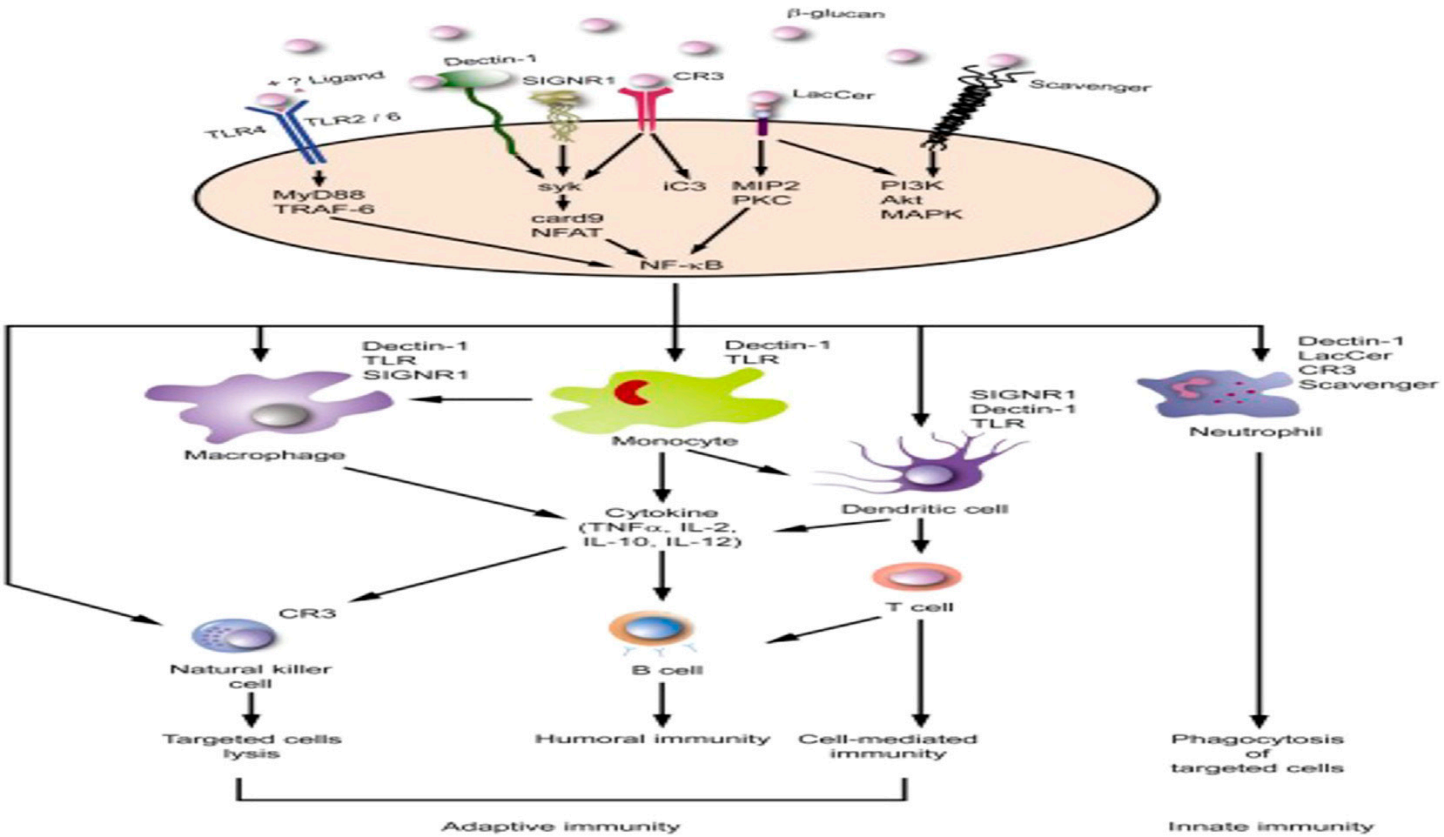
Activation induction of immune system by β-glucans (Rasjidi and Susanto, 2015).

Immunomodulatory activity remains one of the essential features of any drug formulation that makes it effective against any target pathogenic organisms including viral and bacterial diseases. Immunomodulators are substances furnished with the clinical efficacy for altering host responses in the therapy of viral and bacterial infections and have become indispensable agents in relieving the pathology that accompanies viral infections (Kak et al., 2012; Labro, 2012; Zapater et al., 2015; Malemud, 2018). Different bioactive agents from *G. lucidum* are immunomodulators that play significant roles in boosting the immune system, which is the first barrier against infectious diseases (El et al., 2013). Many of these *G. lucidum* bioactive components have been studied for many years for their effects on boosting immune responses and treating infectious (Guggenheim et al., 2014; Mallard et al., 2019; Shao et al., 2019). A typical example is B-D-Glucans, a principal component of Polysaccharide that binds to serum-specific proteins such as Dectin-1, Complement receptor 3 (CR3), Lactosylceramide receptor, Toll-like receptor (TLR), and Scanvenger receptor (Rasjidi and Susanto, 2015). The binding stimulates immune effector cells (T cells, cytotoxic T lymphocytes, dendritic cells, lymphocytes, macrophages, natural killer cells, and others) Figure 4. The immune effector cells’ activation stimulate increasedcytokines production andexpression of interleukins (IL), tumor necrosis factor-alpha (TNF-α), nitric oxide (NO), interferons (IFN), and antibodies (Figure 4) (Kozarski et al., 2011; Li et al., 2014; Xu et al., 2016). The immunomodulating activities like the stimulation of phagocytic activity, acting as inflammatory mediators, and cytokine production has been ascribed to the presence of polysaccharides, especially beta-glucan (Wasser, 2002; Brown et al., 2003; Vetvicka et al., 2008). Furthermore, the potential immunomodulatory and anti-infective activity of terpenes and terpenoids and many more have been established (Jeong et al., 2008; Ma et al., 2011; Su et al., 2020).

### Anti-HIV activity

Acquired immunodeficiency syndrome (AIDS), caused by the Human immunodeficiency virus is highly contagious and affects a great number of people all over the world. The inhibition of HIV progression by ganoderic acids such as ganoderic acid beta, GA-A, and GA-B has been documented (Paydary et al., 2013). In addition, a significant anti-human immunodeficiency virus protease activity with half-maximal inhibitory concentration values of 20–90 Millimole per milliliter exhibited by these ganoderic acids has been reported (Singh et al., 2005; Piraino, 2006). Recently, Kang et al. (2015) also confirmed that ganoderic acid B possesses outrageous inhibiting activity against HIV protease. Furthermore, Zhang et al. (2011), established the inhibition of HIV-1 reverse transcriptase by *G. lucidum extracts*. All these suggested high ganoderic acid activity for HIV management or treatment.

### Antimicrobial activity

Antimicrobial activities of G*L against different* viral, bacterial, and fungal *pathogens have been widely reported with some outstanding results* (Gao et al., 2003; Keypour et al., 2008; Jonathan and Awotona, 2010; Hernández-Márquez et al., 2014; Mehta, 2014; Shah et al., 2014; Zhang et al., 2014; Basnet et al., 2017).

### Anti-malaria

Malaria is a disease caused by some species of Plasmodium and can be successfully treated with artemisinin. Artemisinin resistance in *Plasmodium falciparum* has been reported by Dondorp et al. (2009). Lakornwong et al. (2014) evaluated the anti-malaria properties of triterpene isolated from the mycelium of Ganoderma against *P. falciparum.* The *in vitro P*. *falciparum* examination showed that GA-F and schisanlactone B caused half of *P. falciparum* death with the dose ranging from 6.0 to 10.0 μmol/L.

### Antitumor activity

Ganoderic acids (GAs), a subset of triterpenes have been reported *in vitro* to have antitumor activity, which triggered the apoptosis in cervical carcinoma cells (Toth et al., 1983; Jiang et al., 2008). Human immune system function has been greatly enhanced by GAs (Paterson, 2006). Yuen and Cao (2008) found that GAs prevents the continued division of human cervical carcinoma cells by keeping the cell cycle at the G2 phase. Hsu et al. (2008) discovered the growth inhibition of some tumour cell lines and activation of apoptosis in human leukemia HL-60 cells by GA-B. Jedinak et al. (2011) also expressed the blockage of the cell cycle regulatory protein CDC20 by GA-Y, thus division and growth of invasive and metastatic human breast cancer cells were also prevented.

Generally, cancer remains the foremost clinical challenge despite the advancement in the early diagnosis and chemotherapy of cancer (WHO, 2008). GL is a general supplement taken by healthy persons and cancer patients to boost their immunity along with conventional therapies. *Ganoderma* species are rich sources of countless bioactive components, including antitumoral agents (Zaidman et al., 2005; Borchers et al., 2008). Many polysaccharides and triterpenes components of mushrooms exhibit chemopreventive and/or tumoricidal effects as demonstrated by several studies from *in situ* experiments, laboratory animal and human studies (Tomasi et al., 2004; Zaidman et al., 2005; Yuen and Gohel, 2008; Harhaji et al., 2009; Calviño et al., 2010; Calviño et al., 2010; Wachtel-Galor et al., 2011; Deepalakshmi and Mirunalini, 2011; Wu et al., 2016; Bryant et al., 2017; Zhao and He, 2018). GL polysaccharides (GLPs), *Ganoderma* Triterpenes (GTs), and GL extract possess therapeutic effects on cancers such as prostate cancer (Kao et al., 2016), Lung cancer (Chen et al., 2016), glioma (Wang et al., 2018), cancer of the breast (Smina et al., 2017).

Several studies have described the antitumor activity expressed by *G*. *lucidum* being accomplished through the induction of programmed cell death (Harhaji et al., 2009; Calviño et al., 2010; Ferreira et al., 2015). Furthermore, the isolated constituents from the *G*. *lucidum* were earlier described as controllers of autophagy in several human cancer cell lines (Thyagarajan et al., 2010; Hossain et al., 2012; Liang et al., 2012; Oliveira et al., 2014). Similarly, Oliveira et al. (2014) documented the inhibition of human gastric tumor cell line growth by the methanolic extract of *G*. *lucidum* fruiting bodies through a process that involves cellular autophagy. In addition, Reis et al. (2015) confirmed that a methanolic extract of GL induces autophagy instead of reducing the autophagic flux in AGS cells.

### Anti-inflammatory effect

Inflammation is a natural biological reaction to infection as part of the host’s guard and tissue therapy (Lee and Choi, 2018). Wei et al. (2018) reported that *G. lucidum* polysaccharides (GLPs) can check to swell, sustain intestinal homeostasis and normalize the intestinal immunologic barrier activity in mice. GLPs S58, a sulfated form of a polysaccharide from *G. lucidium* can impede the binding of L-selection with the receptor to trigger the complement systems and block the binding of TNF- and INF-Y to their antibodies. GLPsS58 could inhibit all the L-selection, complement, and cytokine-mediated inflammation pathways (Zhang H. et al., 2018; Zhang K. et al., 2018). However, the anti-inflammatory result of *G. lucidum* polysaccharides plays a significant part in the repair of sensitive skin (Yang et al., 2019).

### Antidiabetic components

*G*. *lucidum* has been confirmed to possess antidiabetic components, hypoglycemic mechanisms and compounds account for hypoglycemic properties. These compounds include polysaccharides, proteoglycans, proteins, and triterpenoids (Gao et al., 2004; Ma et al., 2015). Wang et al. (2015) reported the spore powder of *G. lucidum* potential to reduce hyperglycemia by fostering the synthesis of glycogen and safeguarding gluconeogenesis. According to Tian et al. (2019), protein tyrosine phosphatase 1B (PTP1B) was reported as the main pharmacological target in diabetes. Proteoglycan (Fudan-Yueyang-*G. lucidum*) extracted from *G. lucidum* shown with dermic effects, causing an increase in the blood overexpression of PTP1B, improvement of insulin-dependent glycogen synthesis, and a decline in the blood glucose of a mouse. *Gonoderma lucidum* has been documented to have a hypoglycemic effect coupled with the potential to dysregulate the activity of hepatic glucose-regulated enzymes and epididymal. The antidiabetic properties are also due to its high content of leucine (Zhang H. et al., 2018).

### Antiviral potential

Few studies had reported the antiviral potentials of the GL most especially in animals. The anti-influenza activity of GL aqueous extracts using hot water was investigated against the infected mice when administered intranasally and orally, a finite activity in fighting influenza was confirmed in this study (Zhu et al., 2017). The ability of the triterpenoid compounds obtained from *G*. *lucidum* to interfere with the viral particle and limit its adsorption to the host cells was revealed, thereby preventing the EV71 infection (Zhang et al., 2014).

Triterpenoids compounds obtained from *G. lucidum* have been investigated as antiviral substances against several viral pathogens including the human immunodeficiency virus and dengue virus (DENV) which are fatal microbes spread to humans *via* mosquitoes (Martins et al., 2012; Simmons et al., 2012; Akiner et al., 2016) causing both hemorrhagic fever (Taguchi, 2017; Tang et al., 2017) and shock syndrome (Duyen et al., 2017; Oliveira et al., 2018). An *in vitro* study on the ganodermanontriol (one of the potent triterpenoids) has shown that it can inhibit the DENV NS3pro protein, hence, ganodermanontriol could function as a therapy against DENV disease.

Viral protease inhibitors have been confirmed and considered to offer a defensive and therapeutic potential for the treatment of different kinds of emerging diseases of viral origin and others. Inhibition of the specific target/activities in different pathogenic organisms remains one of the significant ways in the discovery, development, and formulation of drugs. *G. lucidum,* a natural bioresources material had been revealed to be endowed with potential bioactive components that has inhibitory ability against different targets in viral infections and other emerging diseases which could serve as a key source of an alternative compound in the formulation/development of drugs. One of the targets is viral protease activity. *G. lucidum* bioactive components with inhibitory effects against HIV-1 protease activity have been reported. Some of these bioactive agents include Ganomycin B with the efficacy of IC50 = 7.5 μg/ml (El-Mekkawy et al., 1998), Ganoderic acid A with the efficacy of IC50 = 430 µM/CC50 > 62.5 µM on normal human fibroblast BJ cells (El-Mekkawy et al., 1998; Min et al., 2001), Ganoderic acid B with the efficacy of IC50 = 140 µM, Ganoderic acid C1 with IC50 = 240 µM efficacy, Ganoderic acid β with IC50 = 20 µM efficacy (Martinez-Montemayor et al., 2019), Ganodermanondiol with IC50 = 90 µM efficacy, Ganodermanontriol with IC50 = 70 µM efficacy (Martinez-Montemayor et al., 2019), Lucidumol B with IC50 = 50 µM efficacy, 3 β-5 α-dihydroxy-6 β-methoxyergosta-7,22-diene with IC50 = 7.8 μg/ml efficacy (Martinez-Montemayor et al., 2019) coupled with ganolucidic acid A, 3 β-5 α-dihydroxy-6 β-methoxyergosta-7,22-diene (El-Mekkawy et al., 1998; Min et al., 2001; Martinez-Montemayor et al., 2019), isolated from *G. lucidum* has been revealed with potential anti-HIV-1 protease inhibitory activity.

An *in vitro* assay revealed a wide range of antiviral activities of triterpenoids obtained from the *G. lucidum* against pathogenic viruses. Some of these viruses include herpes simplex virus types 1 (HSV-1 and HSV-2), influenza A virus (Flu A), vesicular stomatitis virus (VSV), and human immunodeficiency virus (HIV) (El-Mekkawy et al., 1998; Eo et al., 1999) and dengue virus (DENV) NS2B-NS3 protease. The viral protease was inhibited by Ganoderic acid C2 and Ganosporeric acid, a form of triterpenoids obtained from the *G. lucidum* compared to the reference inhibitor 1,8- Dihydroxy-4,5-dinitroanthraquinone (Bharadwaj et al., 2019).

The two bound polysaccharides namely the neutral protein and acidic protein obtained from *G. lucidum* showed a significant antiviral potential against herpes simplex virus types 1 (HSV- 1) and 2 (HSV-2). However, the acidic protein-based bound polysaccharides revealed more activities by blocking both the HSV-1 and HSV-2, binding to Vero cells at doses of 100 and 90 μg/ml respectively, than the neutral protein-based bound polysaccharides at a 50% effective concentration (EC50 of 300–520 μg/ml). Hence, both the HSV-1 and HSV-2 were hindered from entering Vero cells (Eo et al., 2000).

The potential of *G. lucidum* for the treatment of COVID-19 infection was confirmed in a study by Miqdam et al (2020). Some hematological and immunological responses in the patient with coronavirus (COVID-19) were examined by the uptake of *G. luidum*, the studied hematological parameters showed a substantial reduction of COVID-19 malicious effect played by *G. lucidum* Al-Jumaili et al., 2020. Furthermore, the potency of G. lucidum against the SARS-CoV-2 was observed in a research carried out by Yang et al. (2020), a dose-dependent inhibition of this SARS-CoV enzyme was observed in G. lucidum extracts (IC_50_:41.9 pg/ml) when compared to Coriolus *versicolor* with IC_50_:108.4 pg/ml and Sinomenium acutum with IC_50_:198.6 pg/ml. Hence, *G.* lucidum could serve as a modern, novel, and favourable origin rich in natural bioactive compounds with anti-coronavirus potential (El Sheikha, 2022).

### Antioxidant and Antiaging activity

A countless number of research studies have documented a close link betwixt the richness of *G*. *lucidum* in different phytochemical constituents and its antioxidant biological activity (Kozarski et al., 2011; Abdullah et al., 2012; Mehta, 2014; Kan et al., 2015; Zhang et al., 2015). The efficiency of different antioxidant plants to prevent cancers and many chronic ailments has been shown (Collins 2005; Benzie and Wachtel-Galor et al., 2011). Aging and other age-accompanying disorders have been linked to the presence of long-term free radicals and reactive oxygen species (ROS) (Bishop et al., 2015). For this reason, the study of scavenging free radicals and ROS is of great importance specifically in anti-aging research. Following the UVB treatment, the possession of anti-ROS production in fibroblasts by *G*. *lucidum* polysaccharides (GLPs) had been confirmed (Zeng et al., 2017; Lee et al., 2018). An *in vitro* antioxidant properties of different GL components have been documented (Mau et al., 2002; Yuen and Gohel, 2008; Wachtel-Galor et al., 2011. In an *in vitro* study conducted by Ooi and Liu (2000), the efficacy of the protein-bound polysaccharide (PBP) and polysaccharide peptide to perform similar to the endogenous antioxidant superoxide dismutase (SOD) in cancer-bearing animals was demonstrated.

### Liver dysfunction restored

*Ganoderma lucidum* efficacy to reduce liver dysfunction induced by Copper oxide was investigated by Ghareeb et al. (2021). It was affirmed that the application of G. *lucidum* had a substantial role to play, to restore the liver function dysfunction occasioned by the exposure to copper oxide in rats.

### Cardioprotective effects

*G*. *lucidum* possesses α-tocopherol which is responsible for the protection of mitochondria, mitigation of cardiac toxicity, and mitochondrial dysfunction (Sudheesh et al., 2013). The positive effects of ganopoly on coronary heart disease (CHD) patients coupled with a remarkable decrease in blood pressure and serum cholesterol levels by polysaccharide extract of *G*. *lucidum* were also documented by Gao et al. (2004).

### Hepatoprotection

The hepatoprotective potency of GL most especially the polysaccharides (GLPs) and triterpenoids (GLTs) has been extensively investigated (Yang et al., 2019). Liu et al. (2015) showed the hepatoprotective potency of *G*. *lucidum* polysaccharides on the hepatocyte injury. This is done by the inhibition of lipid peroxidation, and elevation of the antioxidant enzyme activity, alongside the suppression of apoptosis and immune-inflammatory response. Furthermore, GLTs cause considerable cyto-protection against the oxidative damage occasioned by tertbutyl hydrogen peroxide (t-BHP) in hepatocellular carcinoma cells by reducing the level of malondialdehyde and escalating the concentration of glutathione and superoxide dismutase (SOD) (Wu et al., 2016). The liver-protecting effects of the ethanolic extract of *G*. *lucidum* were confirmed according to the histopathology and serum enzymes analysis in mice. Hence, it was concluded that *G*. *lucidum* could ameliorate alcohol-induced liver injury (Zhao et al., 2019). Additionally, *G*. *lucidum* mycelium fermented liquid was detailed with hepatoprotective properties examined in rats (Song et al., 1998).

### Prospect of *G. lucidum* bioactive in the management of emerging diseases in Africa

Emerging diseases are either viral or bacterial in origin but majorly they are of viral origin. The antiviral potential of *G. lucidum* had been reported by several researchers through diverse studies with a few reports from Africa. The ability of this mushroom to be effective against the causative agents has been linked to the presence of triterpenoids and polysaccharides in the mushroom.

*G. lucidum* is considered an unexploited natural source of quite a lot of novel bioactive agents of prodigious value in industry and medicine, especially in drug development in Africa. At present, several bioactive compounds isolated from *G. lucidum* with potent antiviral, antibacterial, antifungal, etc activities against several emerging diseases are under investigation, and the number of studies is constantly increasing (Gu et al., 2007; Isaka et al., 2007; Linnakoski et al., 2018). Therefore, diverse *G. lucidum* bioactive components could become promising candidates for novel, safe, and readily available therapeutics for the treatment and control of several emerging diseases in Africa in a few years (Shahzad et al., 2020) (Seo and Choi, 2021) (Arunachalam et al., 2022).

Few studies had reported the treatment and control of different emerging infectious diseases using some of the African nutraceuticals and phytomedicine. *G. lucidum* has been established as a potential natural bioresource for the improvement of the human immune system against infections and the treatment of different types of viral infections and other types of emerging infectious diseases of diverse origins. Exploitation and exploration of *G. lucidum* extracts have been examined as an immune booster in a patient with Ebola virus hemorrhagic fevers, due to its constituents like selenium, iron, zinc, crude protein, carbohydrates, and other bioactive components like beta-glucan. Beta-glucan is an immune stimulant and is applied in the treatment of various viral infections (Raut, 2020). These potentials show that *G. lucidum* could serve as a novel and future therapy (nutritional therapy and phytopharmaceuticals) from the natural substance for the management of infectious diseases in Africa, thereby complementing the current management and treatment efforts of diseases in African hospitals.

### *G. lucidum* in nanotechnology: A promising future potential remedy to emerging diseases in Africa

Recent advanced developments in the field of nanotechnology have culminated in the development of many nanomaterials. Nanotechnology relies on the ability to design, fabricate, manipulate, study, manufacture, and application of materials at the nanoscale (10^−9^ m) (Sousa et al., 2020). These materials are called nanomaterials which are recently utilized in health maintenance, electronics, cosmetics, and other research areas. Conversely, nanobiotechnology involves the amalgamation of biotechnology and nanotechnology which established that technology on the nanoscale can be practically incorporated with the biology of materials, and mushroom technology is a recent exploring area (Adebayo et al., 2021a,c). The major thrust of nanotechnology is the synthesis of nanoparticles using either physical, chemical, or biological means (green chemistry).

However, the green approach (biological) has been confirmed as the safest method of nanoparticle synthesis which applies microorganisms, plants, animals, and their metabolites for their environmentally safe and eco-friendliness with immense potential (Adelere and Lateef, 2016; Lateef et al., 2016a). Several biological materials have been utilized to synthesize nanoparticles including fungi (Verma et al., 2010), mushrooms (Owaid et al., 2017), bacteria (Lateef et al., 2016b; Oladipo IC. et al., 2017; Oladipo I. C. et al., 2017), plants (Oke et al., 2021; Adebayo et al., 2021a,b,c; Adebayo et al., 2019a, Adebayo et al., 2019b), enzymes (Elegbede et al., 2020) and metabolites obtained from arthropods (Lateef et al., 2016c).

Interestingly, nanofabrication of nanoparticles using fungi has received lots of attention when compared to any other biological materials, due to some unusual advantages and an outstanding property of fungi in nanobiotechnology when used as bio-factories for nanoparticles production. Production of both the intracellular and extracellular enzymes acting as reducing agents for the fabrication of metal nanoparticles has remained an outstanding property of fungi that makes them uniquely suitable for nanoparticle synthesis. Myconanotechnology, a connection linking nanotechnology and mycology is a field with significant prospects, partly owing to the large number and diversity of fungi (Hanafy, 2018; Sousa et al., 2020). Lately, fungi, majorly mushroom system has been revealed as crucial bio-nanofactory for diverse nanoparticles synthesis. An important production of nanoparticles with distinct dimensions and monodispersity was reported by Guilger-Casagrande and Lima (2019).

Mushrooms, and other fungi, are of great importance in nanobiotechnology owing to their potential to produce several beneficial biomolecules which are vital tools for nanoparticle synthesis. The mushroom extracellular production of biomolecules saves the cost of the downstream process while their bioaccumulation and high wall-binding ability have been ascribed to their protein capping and amide linkages in most mushroom-mediated nanoparticles (Anthony et al., 2013). Diverse nanoparticles had been synthesized using several mushrooms coupled with different applications including nanomedicine (Chatterjee et al., 2020), water purification (Mohmed et al., 2017), antimicrobial (Hulikere and Joshi, 2019; Aygün et al., 2020).

Ganoderma has been broadly investigated with a great application as antioxidant, antitumor, antidiabetic, anticancer, antimicrobial, hepatoprotective, anti-inflammatory, anti-HIV, and antiproliferative to mention a few. The most frequent species like *G*. *lucidum*, *G. applanatum* and *G. tsugae,* and *G. capense* have been investigated for their application in biomedicine (Mohanta et al., 2016). A high significant antimicrobial property against different pathogenic bacteria had been shown by *Ganoderma applanatum*-mediated AgNPs. Moreover, Aygün et al. (2020) reported the antimicrobial potential of the AgNPs synthesized by both intracellular and extracellular parts of *G. lucidum* with size 9–21 nm and spherically shaped against various microorganisms including *P. aeruginosa, E. coli*, *C. albicans*, *E. hirae*, *L. pneumophila* and many more.

The biomedical applications of AgNPs mediated by G. *lucidum* concerning drug-resistance of *E. coli* isolated from CAUTI were investigated by Al-Ansari et al. (2020), and it was affirmed that the biosynthesized AgNPs using ethanolic extract of a *G*. *lucidum* demonstrated exceptional biomedical properties. It was further stated that the existence of l,4- Dioxane-2,3-diol, Ethyl acetoacetate ethylene acetal, and Pyridin- 3-ol in the *G. lucidum* is responsible for the observed anti-tumor, antioxidants, and antimicrobial potential of the silver nanoparticles. The result provides a piece of valuable application information for a novel nanoparticle that can be eco-friendly and cost-effective to combat drug resistance in microbes and cancer.

Elumalai et al. (2021) studied the cytotoxicity of the efficacy of the bio-fabrication of gold nanoparticles by using *G. lucidum* against colon cancer cell lines in humans (HT-29). The synthesized nanoparticles were spherical, oval, and irregularly shaped with a size range from 1 to 100 nm while the findings from MTT of biosynthesized nanoparticles revealed potent cytotoxic activity on HT-29 colon cancer cells. Hence, the biosynthesized nanoparticles serve as an effective agent for cancer therapy.

Antioxidant and antibacterial activity estimation of the biosynthesized AgNPs using *Ganoderma lucidum* was conducted by Poudel et al. (2017). A high significant amount of antioxidant and antibacterial activities against human pathogenic bacteria were shown by the AgNPs compared to gentamicin and streptomycin (standard).

The synergy between the tetracycline and the AgNPs (2 nm spherical) produced by G. *lucidum*, showed an enhanced tetracycline with a well-improved antimicrobial activity against different microorganisms (Karwa et al., 2011; Ekar et al., 2015). In the same vein, the antimicrobial potency of the *G. applanatum*-mediated AgNPs with size 133 nm and spherical shape against diverse microorganisms including *S. aureus, E. coli, S. epidermidis, Vibrio cholerae, B. subtilis,* and so on have been documented (Mohanta et al., 2016).

The theranostic applications of the biosynthesized silver nanoparticles (AgNPs) using *G. lucidum* extract were examined by Nguyen et al. (2021a). The susceptibility of the cancer cells to AgNPs/GL with IC50 value of 21:85 μg ml^−1^ for HepG2 and 67:77 μg ml^−1^ for MCF-7 were observed as the antimetastatic impact of AgNPs on the HepG2 and MCF-7 cell lines. Many other researchers including Yu et al. (2019), Nguyen et al. (2021b), and Mohanta et al. (2016) to mention a few worked on biosynthesized nanoparticles using G. *lucidum* with different applications most especially in medical science as a therapy for diverse ailments.

Given all these applications, nanoparticles synthesized using *G. lucidum* have shown great potential in application in diverse areas than free *G. lucidum* solution. Hence, G. *lucidum* in nanotechnology could be a preferable future solution to several emerging diseases in Africa.

## Conclusion

Having established the high content of bioactive compounds present in *Ganoderma* species, especially *G. lucidium*, it can be concluded that it is a mushroom that is worth all the attention it has been getting over the years and recently. It is now abundantly clear why a lot of attention has been given to this mushroom in China where traditional medicine has been solidly established over the years. The wide range of diseases that these bioactive compounds can be used to treat or managed points to the huge potential of this mushroom for the discovery of constituents or drugs that can be used to combat many emerging or reemerging diseases in Africa where the mushroom is known to be indigenous to. It can be concluded that it is the most useful mushroom when it comes to searching or prospecting for bioactive compounds to combat any disease in the world.

### Recommendations

It is recommended that more attention be given to these mushrooms by researchers in Africa. Grants should be made available by governments of African nations and donor countries and agencies for researchers to stimulate their interest in domestication and utilization of Ganoderma mushrooms. Awareness programs on this mushroom should be organized for microbiologists, nutritionists, mycologists, farmers, trado-medical practitioners, pharmacists, and researchers, in general, to educate and discuss the potential of this mushroom in detail.
